# Bisphosphonate-associated atypical fracture of the femur: Spontaneous healing with drug holiday and re-appearance after resumed drug therapy with bilateral simultaneous displaced fractures — a case report

**DOI:** 10.3109/17453674.2011.581267

**Published:** 2011-07-08

**Authors:** Ken Lee Puah, Mann Hong Tan

**Affiliations:** Department of Orthopaedic Surgery, Singapore General Hospital, Singapore

Our patient is a 64-year-old Chinese woman with a history of asthma with previous prednisolone usage, diabetes mellitus, and breast cancer—for which left simple mastectomy and axillary clearance was performed. Her menopause had been at 54 years and she had been on alendronate for 1 year for osteopenia, diagnosed by bone mineral density scan.

She was referred to our department 1 month after her mastectomy for bilateral thigh pain that had lasted 2 weeks, to exclude metastases before commencing chemotherapy. The thighs were tender bilaterally. Plain radiographs of her femurs showed lateral cortex thickening bilaterally with a stress fracture of her right femoral shaft (Figure 1). She had increased uptake at the lower one-third of her right femur and the proximal one-third of her left femur on Tc-99m bone scan.

**Figure 1.**
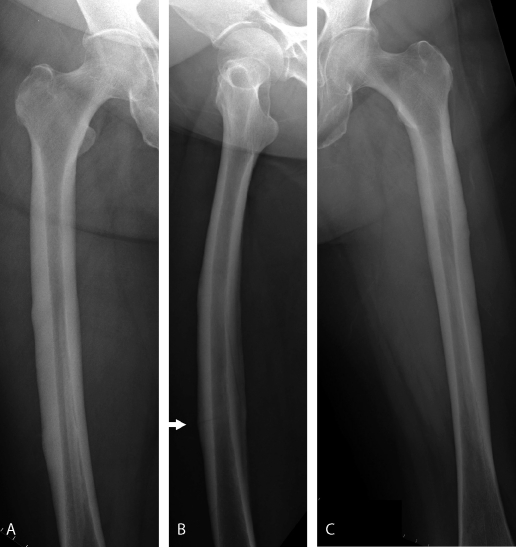
Right (A) and left (C) femur showing lateral cortex thickening. Right femur also showing an undisplaced fracture (B; arrow).

In view of the above findings, she was advised to stop alendronate and was wheelchair-mobilized for 6 weeks. The option of intramedullary nailing for the right femur stress fracture and prophylactic nailing of the left femur was discussed with the patient, but on follow-up, there was radiographic evidence of fracture healing (Figure 2).

**Figure 2.**
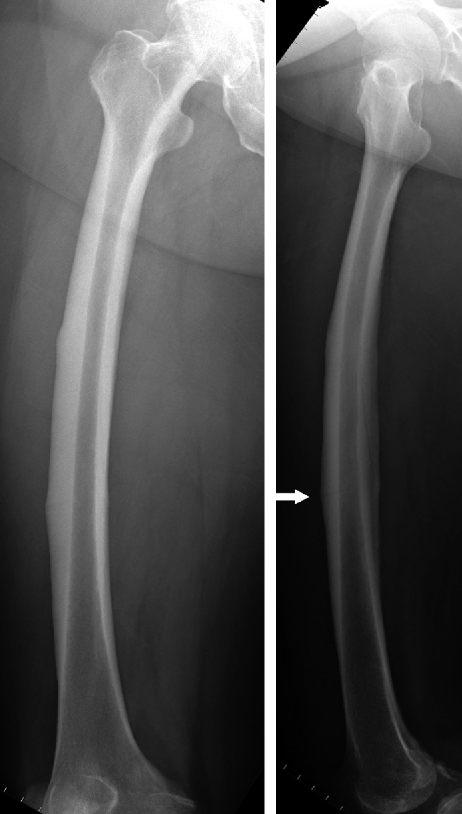
Right femur at 3-month follow-up showing a healing fracture (arrow).

Bone mineral density scan at 3 years since stopping alendronate was normal. Repeat Tc-99m bone scan showed reduced uptake of tracer at the right femur compared to the previous bone scan, consistent with a healing fracture. Apart from occasional bilateral thigh pain, our patient was otherwise well. She was converted to letrazole with ibandronic acid by her oncologist at the 5-year follow-up. Once again, she complained of bilateral aching thigh pain on walking and standing, with difficulty climbing stairs. Plain radiographs of her femurs did not reveal any new fractures.

She was still ambulating until 7 months after starting ibandronate, when she fell in the toilet after a sudden onset of severe bilateral thigh pain; she was brought to the emergency department with bilateral thigh pain and deformity. Plain radiographs of both her femurs showed bilateral shaft fractures (Figure 3). Blood biochemistry showed a normal serum alkaline phosphatase level, 41 U/L (32–103), and normal total serum calcium, 2.11 mmol/L (2.10–2.60). Her serum Ca 15-3 level, a tumor marker for breast carcinoma, was not elevated either.

**Figure 3.**
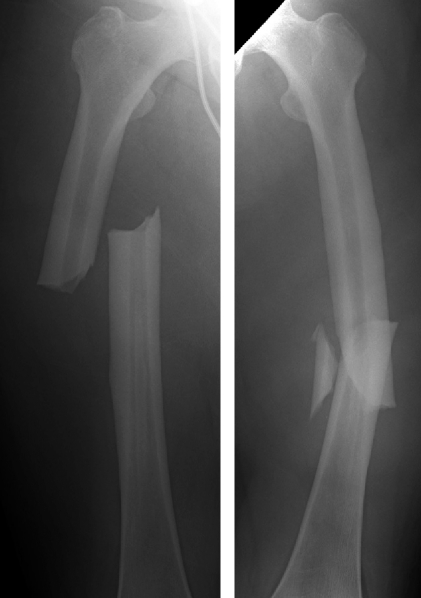
Fractures of the left and right femurs.

Plate fixation of both femoral shafts was performed in a single operation instead of intramedullary nailing with a risk of fat embolism, in view of her history of asthma with baseline hypoxemia (Figure 4). She was wheelchair-mobilized again after surgery and histology from both fracture sites showed fracture hematoma with no evidence of malignancy. The fractures healed uneventfully.

**Figure 4.**
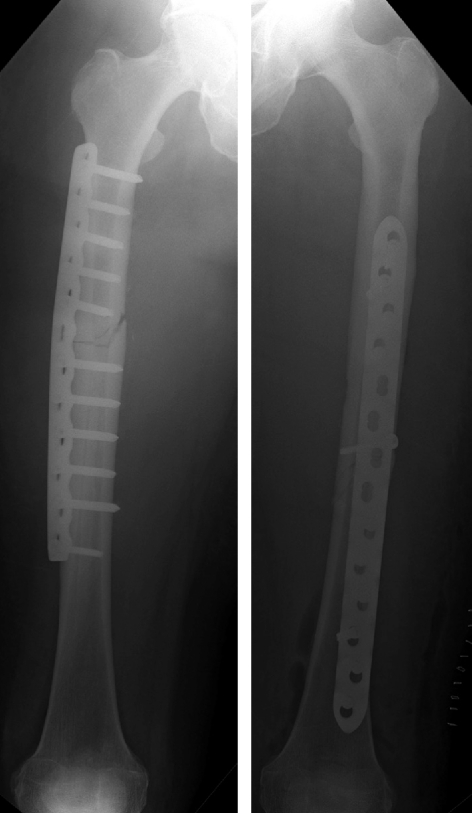
After plate fixation.

## Discussion

The calculated incidence of bisphosphonate-associated femoral diaphyseal fractures has been estimated to be 1 in 1,000 patients per year (Schilcher and Aspenberg 2009). These fractures have been described in patients receiving alendronate for 5–7 years (Kwek et al. 2008, Lenart et al. 2009). Authors have recommended plain radiographs of symptomatic patients to check for lateral femoral cortex thickening and “beaking” with a short oblique or transverse fracture line such as in our patient (Kwek et al. 2008, Lenart et al. 2009). Our patient had received alendronate for only 2 years, yet had evidence on plain radiographs of the aforementioned changes related to its use and with its associated prodromal symptoms. Similar fracture patterns have been reported in patients who were not on bisphophonate therapy (Tan et al. 2010).

The described half-life of alendronate is 10 years and its continued effect after cessation of therapy is why a “drug holiday” works in selected patients who respond to therapy with normal bone mineral density scans after treatment (Khan et al. 1997, Black et al. 2006). Our patient had a normal bone mineral density scan 3 years after stopping alendronate therapy. Despite stopping alendronate for 5 years, she still developed bilateral femur shaft fractures within a year of starting ibandronic acid. This would suggest that the risk of an atypical femur fracture is increased by concurrent use of bisphosphonate rather than being due to accumulation of bisphosphonate in the bones from previous use. In beagles, the effect of suppressed bone turnover by bisphosphonates has been reported to include increased bone strength, but with accumulation of microdamage and a reduction in energy absorption capacity and toughness (Mashiba et al. 2001). Microdamage accumulation has been proposed to contribute to the fragility of osteoporotic bone (Burr et al. 1997). Such microdamage has been suggested to act as a stimulus for targeted bone remodeling through death of osteocytes, resulting in a further increase in microdamage; this gives rise to stress fractures such as our patient's atypical femur fractures (Heino et al. 2009).
